# Genetic Parameters for First Lactation and Lifetime Traits of Nili-Ravi Buffaloes

**DOI:** 10.3389/fvets.2021.557468

**Published:** 2021-06-16

**Authors:** Pooja Tamboli, Anurag Bharadwaj, Amit Chaurasiya, Yogesh Chandrakant Bangar, Andonissamy Jerome

**Affiliations:** ^1^ICAR-Central Institute for Research on Buffaloes, Hisar, India; ^2^ICAR-Indian Grassland and Fodder Research Institute, Jhansi, India; ^3^Nanaji Deshmukh Veterinary Science University, Jabalpur, India; ^4^Lala Lajpat Rai University of Veterinary and Animal Sciences, Hisar, India

**Keywords:** buffalo, Nili-ravi, genetic, phenotypic, traits, association, heritability

## Abstract

The data on first lactation and lifetime performance records of 501 Nili-Ravi were collected for a period from 1983 to 2017 (35 years) maintained at ICAR-Central Institute for Research on Buffaloes, Sub-Campus, Nabha, Punjab. The data were analyzed to calculate heritability, genetic and phenotypic correlation for first lactation traits, viz., Age at First Calving (AFC), First Lactation Total Milk Yield (FLTMY), First Lactation Standard (305 days or less) Milk Yield (FLSMY), First Peak Milk Yield (FPY), First Lactation Length (FLL), First Dry Period (FDP), First Service Period (FSP) and First Calving Interval (FCI), Herd Life (HL), Productive Life (PL), Productive Days (PD), Unproductive Days (UD), Breeding Efficiency (BE), Total Lifetime Milk Yield (Total LTMY), Standard Lifetime Milk Yield (Standard LTMY), Milk Yield Per Day of Productive Life (MY/PL), Milk Yield Per Day of Productive Days (MY/PD), and Milk Yield Per Day of Herd Life (MY/HL). For estimation of variance component and heritability separately for each trait, the uni-trait animal model was equipped, whereas to estimate genetic and phenotypic correlations between traits, bi-trait animal models were fitted. The estimates of heritability for production and reproduction traits of Nili-Ravi were medium, i.e., 0.365 ± 0.087, 0.353 ± 0.071, 0.318 ± 0.082, 0.354 ± 0.076, and 0.362 ± 0.086 for FLSMY, FDP, FSP, FCI, and AFC, respectively. The estimates of heritability were low, i.e., 0.062 ± 0.088, 0.123 ± 0.090, 0.158 ± 0.090, 0.155 ± 0.091, and 0.129 ± 0.091 for HL, PL, PD, Total LTMY, and Standard LTMY and high, i.e., 0.669 ± 0.096 for BE. Genetic correlation for FLTMY was high with FLL (0.710 ± 0.103), and genetic correlation of FLTMY was high and positive with HL, Total LTMY, MY/PL, and MY/PD while low and positive with PL. Genetic correlation of AFC was low and negative with PL, PD, UD, BE, Total LTMY, Standard LTMY, MY/PL, and MY/PD and negative with MY/HL. Significant positive phenotypic association of FPY was seen with FLTMY, FLSMY, FLL, AFC, HL, Total LTMY, and Standard LTMY. Higher heritability of first lactation traits especially FPY suggests sufficient additive genetic variability, which can be exploited under selection and breeding policy in order to improve overall performance of Nili-Ravi buffaloes.

## Introduction

India is ranked first in the world with 176.3 million tons of milk production, and most of the production (49%) was contributed by buffaloes. The per capita availability of milk up to 375 g/day was achieved in recent time (1). Buffaloes are present almost in all parts of the country with varying population density, the majority being concentrated in the North and Western parts. Nili-Ravi, a buffalo breed, is found in the state of Punjab and largely distributed in the belt of Ravi River, having its concentration in Amritsar, Tarn Taran, and Ferozepur districts (2). Lifetime performance of animal especially depends on different production and reproduction traits of earlier lactations, which determine genetic worth of the buffaloes. Hence, the lifetime traits can be improved by the selection of animals on the basis of traits of earlier lactations through correlated response. Assessment of genetic progress attained by the selection programs gives an idea about advantages of breeding program and to incorporate required adjustments.

The traits having high heritability can be efficiently improved through appropriate selective breeding. The heritability provides an opportunity to forecast the outcome of breeding strategy. High heritability observed for the First Lactation Total Milk Yield (FLTMY) and First Lactation Standard (305 days or less) Milk Yield (FLSMY) in Nili-Ravi (3) indicates that there is enough scope of the traits to respond in the next generation. Reduction in Age at First Calving (AFC) is associated with improvement in the lifetime milk yield, herd life, productive life, and productive days in buffaloes (4). For economizing milk production, it is desirable to decrease AFC (5). Early first calving of animals would result in longer productive life (6). Thus, dairymen have a considerable concern in the relationship of different production and reproduction parameters with lifetime traits. Framing for profitable selection and breeding systems requires knowledge of genetic and phenotypic parameters of early and lifetime performance traits to enhance genetic potential of buffaloes. Information regarding genetic and phenotypic relationship between first lactation and lifetime traits in Nili-Ravi buffaloes is scarce. Therefore, the present study was designed to evaluate heritability, genetic and phenotypic correlation for first lactation and lifetime performance traits in Nili-Ravi buffaloes.

## Materials and Methods

### Study Location, Collection of Data, and Its Classification

The present study was carried out on the performance records of Nili-Ravi buffaloes (*n* = 501) maintained at ICAR-Central Institute for Research on Buffaloes, Sub-Campus, Nabha, Punjab. The data on first lactation and lifetime performance records of study animals that remained in the herd for at least three lactations and disposed were collected from the history sheet of animal for a period from 1983 to 2017. The data for all the buffaloes born and calved at the farms were recorded up to the date of death/culling. The animals having minimum lactation length >150 days and production >1,000 kg for first lactation were considered. Incomplete and abnormal records due to abortion, stillbirth, chronic illness, etc., were excluded.

Traits analyzed in our study were first lactation traits, viz., AFC in months, FLTMY in kg, FLSMY in kg, First Peak Milk Yield (FPY) in kg/day, First Lactation Length (FLL) in days, First Dry Period (FDP) in days, First Service Period (FSP) in days, First Calving Interval (FCI) in days, Lifetime performance traits such as Herd Life (HL) in days, Productive Life (PL) in days, Productive Days (PD) in days, Unproductive Days (UD) in days, Breeding Efficiency (BE) in %, Total Lifetime Milk Yield (Total LTMY) in kg, Standard Lifetime Milk Yield (Standard LTMY) in kg, Milk Yield Per Day of Productive Life (MY/PL) in kg/day, Milk Yield Per Day of Productive Days (MY/PD) in kg/day, and Milk Yield Per Day of Herd Life (MY/HL) in kg/day.

The herd life was considered the duration from birth to disposal of animal. Productive life was defined as total days from date of first calving to date of last dry or date of disposal if animal is in lactation. Productive days were the sum of the number of days in milk in different lactations in the same herd. Unproductive days were the sum of dry periods in different lactations in the same herd. Lifetime milk yield was defined as total amount of milk produced by a buffalo from the initiation of first lactation until last day in milk in the herd. It was taken for those animals that remained in the herd for at least three lactations. Milk yield per day of productive life was measured as lifetime milk yield divided by the productive life. Milk yield per day of productive days was measured as lifetime milk yield divided by the productive days. Milk yield per day of herd life was measured as lifetime milk yield divided by herd life (7, 8).

### Statistical Analysis

The data were analyzed to calculate heritability, genetic and phenotypic correlation for first lactation, and lifetime traits. Data were initially analyzed by least squares analysis model (SPSS Statistics 20.0 software) to identify the significant fixed effects. The least squares model for AFC included season of birth (Level 4) and period of birth (Level 6) as fixed effects. Fixed effects for first lactation and lifetime traits were AFC (Level 6), season of first calving (Level 4), and Period of first calving (Level 6). Lactation completed (Level 5) was also included along with other fixed effects for lifetime traits. The statistical significance was tested at 5% and 1% level. Only significant effects under least squares analysis for each trait were included under univariate animal model for estimating heritability and associations between AFC, first lactation traits, and lifetime productivity (9, 10).

The estimates of variance components and genetic parameters were obtained *via* Average Information-Restricted Maximum Likelihood (REML) using Wombat software (11). The convergence was assumed if the difference in log likelihood function between consecutive iterations was lower than 5 × 10^−4^.

The univariate animal model consisting of significant fixed effects and random animal effects was used in this study as follows:

$$\begin{array}{l} y=X\beta +Zu+e \end{array}$$

where y is the vector of records for different traits (FLTMY, Total LTMY, etc.)

X is the incidence matrices assigning observations to fixed effects

β is the vector of fixed effects

Z is the incidence matrices assigning observations to random effects

u is the vector of random animal effects

e is the vector of random residual effects (error) distributed normally with mean zero and variance σ^2^

Furthermore, bivariate analysis was performed between each pair of the first lactation and lifetime traits to estimate the genetic and phenotypic correlations (12). The significance of phenotypic correlation was tested using “*t*-test” at 5 or 1% level.

## Results

### Heritability Estimates for First Lactation Production and Reproduction Traits

Heritability estimates are helpful for the prediction of genetic response and accuracy in selection (5). The estimates of heritability for production and reproduction (Table 1) traits of Nili-Ravi were medium for FLSMY (0.365 ± 0.087), FDP (0.353 ± 0.071), FSP (0.318 ± 0.082), FCI (0.354 ± 0.076), and AFC (0.362 ± 0.086); moderate for FLTMY (0.439 ± 0.091) and FLL (0.546 ± 0.065); and high for FPY (0.640 ± 0.112).

**Table 1:** Heritability (diagonal), genetic (above diagonal), and phenotypic (below diagonal) correlation among first lactation traits.

**Traits**	**FLTMY (kg)**	**FLSMY (kg)**	**FPY (kg/day)**	**FLL (days)**	**AFC (months)**	**FDP (days)**	**FSP (days)**	**FCI (days)**
FLTMY (kg)	**0.439**, **0.091**	0.421, 0.149	0.361, 0.153	0.710, 0.103	0.164, 0.208	−0.377, 0.185	0.359, 0.218	0.319, 0.198
FLSMY (kg)	0.687, 0.026^**^	**0.365**, **0.087**	0.562, 0.144	0.026, 0.133	0.442, 0.227	0.491, 0.240	0.483, 0.252	0.445, 0.226
FPY (kg/day)	0.459, 0.040^**^	0.573, 0.035^**^	**0.640**, **0.112**	−0.309, 0.150	0.201, 0.193	−0.089, 0.182	−0.215, 0.216	−0.356, 0.201
FLL (days)	0.564, 0.026^**^	0.131, 0.035^**^	0.108, 0.041^*^	**0.546**, **0.065**	0.022, 0.158	−0.357, 0.151	0.512, 0.131	0.597, 0.116
AFC (months)	0.142, 0.042^**^	0.210, 0.041^**^	0.107, 0.046^*^	0.008, 0.036	**0.362**, **0.086**	0.091, 0.230	0.065, 0.261	0.098, 0.233
FDP (days)	−0.259, 0.037^**^	0.017, 0.038	−0.025, 0.043	−0.214, 0.03^**^	0.023, 0.038	**0.353**, **0.071**	0.581, 0.134	0.536, 0.128
FSP (days)	0.179, 0.041^**^	0.098, 0.041^*^	−0.042, 0.046	0.488, 0.027^**^	0.009, 0.041	0.728, 0.021^**^	**0.318**, **0.082**	0.963, 0.020
FCI days)	0.188, 0.039^**^	0.110, 0.039^*^	−0.100, 0.044^*^	0.543, 0.026^**^	0.025, 0.039	0.704, 0.021^**^	0.981, 0.003^**^	**0.354**, **0.076**

*Significant at*

* *p < 0.05*,

** *p < 0.01*.

*AFC, Age at First Calving; FLTMY, First Lactation Total Milk Yield; FLSMY, First Lactation Standard (305 days or less) Milk Yield; FPY, First Peak Milk Yield; FLL, First Lactation Length; FDP, First Dry Period; FSP, First Service Period; FCI, First Calving Interval*.

*Bold values represent Heritability*

### Heritability Estimates for Lifetime Performance Traits

The heritability estimates for lifetime performance traits of Nili-Ravi are presented in Table 2. In Nili-Ravi, the estimates of heritability were low for HL (0.062 ± 0.088), PL (0.123 ± 0.090), PD (0.158 ± 0.090), Total LTMY (0.155 ± 0.091), and Standard LTMY (0.129 ± 0.091); medium (0.2–0.4) for UD (0.228 ± 0.089), MY/PL (0.354 ± 0.094), MY/PD (0.231 ± 0.086), and MY/HL (0.261±0.089); and high for BE (0.669 ± 0.096).

**Table 2:** Heritability (diagonal), genetic (above diagonal), and phenotypic (below diagonal) correlation among lifetime traits.

**Traits**	**HL (days)**	**PL (days)**	**PD (days)**	**UD (days)**	**BE (%)**	**Total LTMY (kg)**	**Standard LTMY (kg)**	**MY/PL (kg/day)**	**MY/PD (kg/day)**	**MY/HL (kg/day)**
HL (days)	**0.062** **±** **0.088**	0.304 ± 0.197	0.611 ± 0.219	−0.439 ± 3.447	−0.229 ± 0.471	0.750 ± 0.239	0.529 ± 0.261	0.529 ± 0.504	0.298 ± 0.403	0.483 ± 0.332
PL (days)	0.893 ± 0.014^**^	**0.123** **±** **0.090**	0.887 ± 0.103	0.562 ± 0.217	−0.305 ± 0.322	0.676 ± 0.200	0.815 ± 0.191	−0.355 ± 0.594	−0.384 ± 0.906	0.578 ± 0.251
PD (days)	0.890 ± 0.014^**^	0.969 ± 0.008^**^	**0.158** **±** **0.090**	0.117 ± 0.191	−0.126 ± 0.284	0.868 ± 0.130	0.867 ± 0.154	−0.005 ± 53500	−0.233 ± 0.866	0.717 ± 0.195
UD (days)	0.704 ± 0.026^**^	0.847 ± 0.018^**^	0.689 ± 0.027^**^	**0.228** **±** **0.089**	−0.431 ± 0.215	−0.101 ± 2.241	0.202 ± 0.249	−0.755 ± 0.334	−0.409 ± 0.471	−0.042 ± 1.552
BE (%)	−0.046 ± 0.044	−0.143 ± 0.044^**^	−0.066 ± 0.044	−0.278 ± 0.042^**^	**0.669** **±** **0.096**	0.063 ± 0.279	0.059 ± 0.278	0.272 ± 0.173	0.045 ± 0.190	0.120 ± 0.209
Total LTMY (kg)	0.865 ± 0.017^**^	0.900 ± 0.015^**^	0.938 ± 0.011^**^	0.621 ± 0.030^**^	0.041 ± 0.045	**0.155** **±** **0.091**	0.936 ± 0.069	0.352 ± 0.266	0.142 ± 0.216	0.788 ± 0.131
Standard LTMY (kg)	0.844 ± 0.018^**^	0.903 ± 0.014^**^	0.923 ± 0.013^**^	0.662 ± 0.029^**^	0.080 ± 0.044	0.985 ± 0.005^**^	**0.129** **±** **0.091**	0.116 ± 0.223	0.018 ± 0.050	0.759 ± 0.148
MY/PL (kg/day)	0.295 ± 0.041^**^	0.157 ± 0.044^**^	0.274 ± 0.041^**^	−0.131 ± 0.044^**^	0.242 ± 0.042^**^	0.477 ± 0.035^**^	0.440 ± 0.036^**^	**0.354** **±** **0.094**	0.703 ± 0.129	0.207 ± 0.198
MY/PD (kg/day)	0.393 ± 0.037^**^	0.308 ± 0.039^**^	0.328 ± 0.038^**^	0.199 ± 0.041^**^	0.087 ± 0.043^*^	0.554 ± 0.030^**^	0.548 ± 0.030^**^	0.638 ± 0.022^**^	**0.231** **±** **0.086**	−0.019 ± 68.352
MY/HL (kg/day)	0.478 ± 0.031^**^	0.568 ± 0.028^**^	0.629 ± 0.024^**^	0.313 ± 0.038^**^	0.074 ± 0.044	0.710 ± 0.017^**^	0.700 ± 0.018^**^	0.417 ± 0.032^**^	0.416 ± 0.030^**^	**0.261** **±** **0.089**

*Significant at*

* *p < 0.05*,

** *p < 0.01*.

*HL, Herd Life; PL, Productive Life; PD, Productive Days; UD, Unproductive Days; BE, Breeding Efficiency; Total LTMY, Total Lifetime Milk Yield; Standard LTMY, Standard Lifetime Milk Yield; MY/PL, Milk Yield Per Day of Productive Life; MY/PD, Milk Yield Per Day of Productive Days; MY/HL, Milk Yield Per Day of Herd Life*.

*Bold values represent Heritability*

### Estimates of Genetic Correlation for First Lactation Performance Traits

Estimates of genetic correlation among production and reproduction traits in Nili-Ravi are presented in Table 1. High and positive correlation for FLTMY with FLL (0.710 ± 0.103), medium and positive with FPY (0.361 ± 0.153), and moderate and positive with FLSMY (0.421 ± 0.149) was observed. Genetic correlation of FLSMY was medium and positive with FPY (0.562 ± 0.144) while low and positive with FLL (0.026 ± 0.133). The correlation between FPY and FLL (−0.309 ± 0.150) was medium and negative. Estimates of genetic correlation were low and positive for AFC with FSP (0.065 ± 0.261), FCI (0.098 ± 0.233), and FDP (0.091 ± 0.230). Genetic correlation of FDP was moderate and positive with FCI (0.536 ± 0.128) and FSP (0.581 ± 0.134). The correlation between FSP and FCI (0.963 ± 0.020) was high and positive.

The genetic correlation between first lactation production and reproduction traits for Nili-Ravi is presented in Table 1. In Nili-Ravi, estimates of genetic correlation (Table 1) were low and positive for FLTMY with AFC (0.164 ± 0.208) and medium and negative with FDP but positive with FSP and FCI. Moderate and positive genetic correlation of FLSMY was noticed with AFC, FDP, FSP, and FCI. Genetic correlation of FPY was low and negative with FDP and medium and positive with AFC but negative with FSP and FCI. The genetic correlation of FLL was low and positive with AFC, medium and negative with FDP, while moderate and positive with FSP and FCI.

### Genetic Correlation Among Lifetime Traits

In Nili-Ravi, estimates of genetic correlation (Table 2) of HL was high and positive with PD and Total LTMY; moderate and positive with Standard LTMY, MY/PL, and MY/HL but negative with UD; medium and positive with MY/PD and PL and negative with BE. Genetic correlation of PL was high and positive with PD, Total LTMY, and Standard LTMY; moderate and positive with UD and MY/HL; and medium and negative with BE, MY/PL, and MY/PD. The correlation of PD was high and positive with Total LTMY, Standard LTMY, and MY/HL; medium and negative with MY/PD; low and positive with UD but negative with BE and MY/PL. The correlation of UD was medium and positive with Standard LTMY; low and negative with Total LTMY and MY/HL; moderate and negative with BE and MY/PD; high and negative with MY/PL. The genetic correlation of BE was low and positive with Total LTMY, Standard LTMY, MY/PD, and MY/HL; medium and positive with MY/PL. Table 2 elucidates that the genetic correlation of Total LTMY was high and positive with Standard LTMY (0.936 ± 0.069) and MY/HL (0.788 ± 0.131); medium and positive with MY/PL; low and positive with MY/PD. The genetic correlation of Standard LTMY was low and positive with MY/PL and MY/PD but high and positive with MY/HL. The correlation of MY/PL was medium and positive with MY/HL but high and positive with MY/PD. The genetic correlation between MY/PD and MY/HL was low and negative.

### Genetic Correlation Between First Lactation Performance and Lifetime Traits

The genetic correlation estimates between first lactation production and reproduction traits with lifetime traits of Nili-Ravi are presented in Table 3. It can be inferred from Table 3 that the genetic correlation of FLTMY was high and positive with HL, Total LTMY, MY/PL, and MY/PD; moderate and positive with Standard LTMY; medium and positive with PD and MY/HL. Hence, selection for one trait simultaneously leads to genetic gain in the other parameters, as the genetic correlation between the traits is higher and positive.

**Table 3:** Genetic correlation between first lactation and lifetime traits in Nili-Ravi buffaloes.

**Traits**	**FLTMY (kg)**	**FLSMY (kg)**	**FPY (kg/day)**	**FLL (days)**	**AFC (months)**	**FDP (days)**	**FSP (days)**	**FCI (days)**
HL (days)	0.887, 0.683	0.323, 0.578	0.074, 0.382	0.834, 0.579	0.330, 0.525	−0.383, 0.649	0.382, 0.635	0.425, 0.599
PL (days)	0.141, 0.384	0.083, 0.373	−0.396, 0.419	0.603, 0.336	−0.108, 0.374	0.234, 0.370	0.673, 0.444	0.746, 0.424
PD (days)	0.374, 0.334	0.015, 0.178	−0.332, 0.357	0.777, 0.291	−0.024, 0.266	−0.137, 0.348	0.480, 0.384	0.585, 0.364
UD (days)	−0.365, 0.273	0.151, 0.322	−0.258, 0.266	−0.096, 0.207	−0.188, 0.288	0.748, 0.241	0.588, 0.290	0.556, 0.266
BE (%)	−0.228, 0.164	−0.366, 0.184	0.244, 0.159	−0.228, 0.115	−0.114, 0.178	−0.645, 0.133	−0.833, 0.125	−0.761, 0.113
Total LTMY (kg)	0.674, 0.325	0.099, 0.271	−0.047, 1.370	0.870, 0.316	−0.106, 0.361	−0.423, 0.359	0.339, 0.396	0.423, 0.370
Standard LTMY (kg)	0.483, 0.359	0.179, 0.322	−0.036, 1.002	0.727, 0.350	−0.164, 0.400	−0.186, 0.384	0.416, 0.453	0.498, 0.426
MY/PL (kg/day)	0.653, 0.161	0.085, 0.177	0.458, 0.175	0.258, 0.167	−0.082, 0.273	−0.653, 0.192	−0.304, 0.243	−0.328, 0.223
MY/PD (kg/day)	0.603, 0.209	0.327, 0.227	0.664, 0.195	0.036, 0.198	−0.182, 0.314	−0.288, 0.263	−0.091, 0.287	−0.215, 0.277
MY/HL (kg/day)	0.363, 0.221	−0.051, 0.785	−0.258, 0.290	0.684, 0.201	−0.261, 0.265	−0.316, 0.255	0.210, 0.296	0.346, 0.275

*For abbreviations, refer to the legends of Tables 1, 2*.

But the correlation of FLTMY was medium and negative with UD and BE while low and positive with PL. The FLSMY was associated positively with HL, PL, PD, UD, Total LTMY, Standard LTMY, MY/PL, and MY/PD but negatively with BE and MY/HL. The genetic correlation of FPY showed that it was low and positive with HL; low and negative with Total LTMY; medium and negative with PL, PD, UD, Standard LTMY, and MY/HL; medium and positive with BE; moderate and positive with MY/PL; high and positive with MY/PD. The FLL was correlated positively with HL, PL, PD, Total LTMY, Standard LTMY, MY/HL, MY/PD, and MY/PL while negatively with UD and BE.

Perusal of Table 3 revealed that in Nili-Ravi, the genetic correlation of AFC was low and negative with PL, PD, UD, BE, Total LTMY, Standard LTMY, MY/PL, and MY/PD; medium and positive with HL; medium and negative with MY/HL. The genetic correlation of FDP was high and positive with UD; medium and positive with PL while moderate and negative with Total LTMY; high and negative with BE and MY/PL; low and negative with PD and Standard LTMY; medium and negative with HL, MY/PD, and MY/HL. The FSP was positively correlated with HL PL PD, UD Total LTMY, Standard LTMY, and MY/HL, whereas it was negative with MY/PL, MY/PD, and BE. The positive association of FCI was found with HL, PL, PD, UD, Total LTMY, Standard LTMY, and MY/HL whereas negative with BE, MY/PL, and MY/PD.

### Phenotypic Correlation for First Lactation Performance Traits

It is apparent from Table 1 that in Nili-Ravi, estimates of phenotypic correlation was high and positive for FLTMY with FLSMY (0.687 ± 0.026); moderate and positive with FPY (0.459 ± 0.040) and FLL (0.564 ± 0.026). The correlation of FLSMY was highly significant (*p* ≤ 0.01) and positive with FPY (0.573 ± 0.035) and FLL (0.131 ± 0.035). The association between FPY and FLL was significant (*p* ≤ 0.05) and positive. Estimates of phenotypic correlation were low and positive for AFC with FDP, FSP, and FCI. Phenotypic association of FDP was high and positive with FCI (0.704 ± 0.021) and FSP (0.728 ± 0.021). The correlation between FSP and FCI (0.981 ± 0.003) was also high and positive.

Perusal of Table 1 indicates that in Nili-Ravi, estimates of phenotypic correlation were highly significant (*p* ≤ 0.01) and positive for FLTMY with AFC, FSP, and FCI but highly significant (*p* ≤ 0.01) and negative with FDP. The correlation of FLSMY was significant (*p* ≤ 0.05) and positive with AFC, FSP, and FCI but non-significant (*p* > 0.05) with FDP. Phenotypic correlation of FPY was significant (*p* ≤ 0.05) and positive with AFC (0.107 ± 0.046) but negative with FCI. The correlation of FLL was highly significant (*p* ≤ 0.01) and negative with FDP but positive with FSP and FCI. No relationship between FLL and AFC was found.

### Phenotypic Correlation Among Lifetime Traits

It is obvious from Table 2 that estimates of phenotypic correlation in Nili-Ravi for HL were high and positive with PL (0.893 ± 0.014), PD (0.890 ± 0.014), UD (0.704 ± 0.026), Total LTMY (0.865 ± 0.017), and Standard LTMY (0.844 ± 0.018); moderate and positive with MY/HL; medium and positive with MY/PD and MY/PL whereas low and negative with BE. The correlation of PL was high and positive with PD, UD, Total LTMY, and Standard LTMY; moderate and positive with MY/HL, medium and positive with MY/PD; low and positive with MY/PL but low and negative with BE. Positive correlation of PD was observed with UD, Total LTMY, Standard LTMY, MY/HL, MY/PL, and MY/PD whereas negative with BE. The association of UD was high and positive with Total LTMY and Standard LTMY; medium and positive with MY/HL; low and positive with MY/PD but low and negative with MY/PL; moderate and negative with BE. Low and positive phenotypic correlation of BE was observed with Total LTMY, Standard LTMY, MY/PD, and MY/HL while medium and positive with MY/PL. Table 2 represents that the correlation of Total LTMY was high and positive with Standard LTMY and MY/HL; moderate and positive with MY/PD and MY/PL. High and positive phenotypic association of Standard LTMY was found with MY/HL (0.700 ± 0.018) but moderate and positive with MY/PL and MY/PD. The correlation of MY/PL was high and positive with MY/PD; moderate and positive with MY/HL. Moderate and positive correlation was seen between MY/PD and MY/HL.

### Phenotypic Correlation Between First Lactation Performance and Lifetime Traits

The phenotypic correlation estimates between first lactation performance and lifetime traits are shown in Table 4 for Nili-Ravi buffaloes. The phenotypic correlation between first lactation production and lifetime traits in Nili-Ravi is presented in Table 4. The association of FLTMY and FLSMY was significant (*p* ≤ 0.05) and positive with HL, PD, Total LTMY, Standard LTMY, MY/PL, MY/PD, and MY/HL while negative with UD and BE. The correlation of FPY was highly significant (*p* ≤ 0.01) and positive with Total LTMY (0.208 ± 0.046), Standard LTMY (0.206 ± 0.046), MY/PL, MY/PD, and MY/HL; significant (*p* ≤ 0.05) and positive with HL (0.113 ± 0.047). The FLL was associated positively with PL, PD, Total LTMY, Standard LTMY, and MY/HL and negatively with UD, BE, and MY/PD.

**Table 4:** Phenotypic correlation between first lactation and lifetime traits in Nili-Ravi buffaloes.

**Traits**	**FLTMY (kg)**	**FLSMY (kg)**	**FPY (kg/day)**	**FLL (days)**	**AFC (months)**	**FDP (days)**	**FSP (days)**	**FCI (days)**
HL (days)	0.143, 0.043^**^	0.087, 0.042^*^	0.113, 0.047^*^	0.080, 0.036	0.135, 0.041^**^	−0.028, 0.039	0.052, 0.041	0.034, 0.040
PL (days)	0.052, 0.044	0.087, 0.043^*^	0.039, 0.048	0.094, 0.036^*^	−0.076, 0.042	0.127, 0.038^**^	0.186, 0.041^**^	0.178, 0.039^**^
PD (days)	0.147, 0.043^**^	0.127, 0.042^**^	0.048, 0.048	0.189, 0.036^**^	−0.055, 0.042	0.011, 0.039	0.147, 0.041^**^	0.147, 0.040^**^
UD (days)	−0.165, 0.043^**^	−0.018, 0.042	0.012, 0.048	−0.131, 0.036^**^	−0.104, 0.042^*^	0.349, 0.035^**^	0.230, 0.039^**^	0.204, 0.038^**^
BE (%)	−0.114, 0.044^*^	−0.127, 0.043^**^	0.073, 0.049	−0.249, 0.034^**^	−0.137, 0.043^**^	−0.568, 0.032^**^	−0.688, 0.029^**^	−0.670, 0.028^**^
Total LTMY (kg)	0.292, 0.041^**^	0.257, 0.041^**^	0.208, 0.046^**^	0.169, 0.036^**^	−0.048, 0.043	−0.066, 0.039	0.067, 0.042	0.066, 0.040
Standard LTMY (kg)	0.226, 0.042^**^	0.267, 0.041^**^	0.206, 0.046^**^	0.104, 0.037^*^	−0.060, 0.043	−0.032, 0.039	0.049, 0.042	0.048, 0.040
MY/PL (kg/day)	0.509, 0.034^**^	0.404, 0.036^**^	0.428, 0.042^**^	0.042, 0.037	0.052, 0.043	−0.289, 0.036^**^	−0.202, 0.041^**^	−0.218, 0.040^**^
MY/PD (kg/day)	0.415, 0.036^**^	0.439, 0.034^**^	0.512, 0.038^**^	−0.140, 0.036^**^	0.023, 0.042	−0.070, 0.038	−0.120, 0.041^**^	−0.161, 0.039^**^
MY/HL (kg/day)	0.316, 0.039^**^	0.272, 0.039^**^	0.118, 0.046^**^	0.248, 0.035^**^	−0.127, 0.041^**^	−0.097, 0.038^*^	0.067, 0.041	0.098, 0.040^*^

*Significant at*

* *p < 0.05*,

** *p < 0.01*.

*For abbreviations, refer to the legends of Tables 1, 2*.

It is clear from Table 4 that in Nili-Ravi, the phenotypic correlation of AFC was positive with HL, MY/PL, and MY/PD and negative with UD, BE, and MY/HL. The correlation of FDP was highly significant (*p* ≤ 0.01) and positive with PL and UD but negative with BE, MY/HL, and MY/PL. The phenotypic association of FSP was highly significant (*p* ≤ 0.01) and positive with PL, PD, and UD and negative with BE, MY/PL, and MY/PD. The FCI was associated positively with HL, PL, PD, UD, Total LTMY, Standard LTMY, and MY/HL and negatively with BE, MY/PL, and MY/PD.

## Discussion

Moderate to high estimates of heritability for various traits show sufficient additive genetic variance for the traits that could be used for selection. In this study, higher estimates of heritability for FPY indicate the extreme reliance of the trait upon the growth rate and other structural traits that commonly have high heritability. Hence, FPY should be given more weightage while selection as the trait is highly heritable and responds in the next generation, which simultaneously also leads to improvement in the lifetime performance. In accordance with our findings, Chaudhari (8) recorded medium heritability for FSP and FCI in Nili-Ravi. Medium heritability for AFC was reported by Kumar et al. (13) in Murrah, Singh and Yadav (14) in Nili-Ravi, and Kumar et al. (15) in cattle. In contrast, high heritability was observed for the FLTMY and FLSMY in Nili-Ravi (3). Also, Chaudhari (8) reported low heritability for FLTMY, FLSMY, FLL, and FDP; medium for FPY; and high for AFC in Nili-Ravi. Higher estimates of heritability, i.e., 0.63 and 0.82, for AFC were observed by Singh and Gurnani (16) in Karan Swiss and Karan Fries cattle, respectively. While lower heritability for AFC was estimated as 0.11 in Mehsana buffaloes by Parmar et al. (5) and 0.03 by Brzakova et al. (17) in Holstein Friesian. The reason for higher heritability of first lactation traits in Nili-Ravi breed under our investigation could be attributed to less inbreeding and more genetic variation. Similar to our results, low heritability for LTMY, PL, and HL was noticed by Bashir et al. (18) in Nili-Ravi buffaloes. Singh et al. (19) also reported low heritability for LTMY in Nili-Ravi. Contrary to our finding, heritability estimated for Total LTMY, PL, and HL were medium while low for UD in Nili-Ravi buffaloes (8). Ali et al. (4) also reported medium heritability estimates of LTMY and HL while moderat for PL.

Strong genetic correlation exists between these reproductive traits; hence, any one of the traits can be used to evaluate reproductive status. A highly positive association reveals the increase in FSP; there is subsequent increment in FDP and FCI as the gestation period and lactation length almost remain constant. Similar to our results, the genetic correlation of FLTMY was positive with FLSMY, FPY, and FLL in Nili-Ravi buffaloes (8). Similarly, highly positive genetic correlation of FDP with FSP and FCI was reported by Godara (20) in Murrah. Also, Chaudhari (8) reported a positive genetic correlation of AFC with FLTMY, FLSMY, FPY, FLL, FDP, FSP, and FCI; FDP with FSP and FCI. The FLSMY had medium positive genetic correlation with FSP and FCI. Sachdeva and Gurnani (21) also reported a positive genetic correlation between FLTMY and FCI in dairy animals. However, our results differ from the findings of Chaudhari (8) who revealed a negative correlation of FLSMY with FDP, and Patil et al. (22) noticed negative relationships of AFC with FSP, FLTMY, and FPY, while AFC was positively correlated with FCI in Murrah.

Highly positive association of productive days with lifetime milk yield confesses the traits are influenced by the same genes. A high correlation among different traits indicates concurrent progression of the traits through selection and genetic improvement to enhance genetic potential and establish herd of superior animals. In agreement to our findings, highly positive genetic correlations among lifetime traits were reported by Kaushik et al. (23) and Singh et al. (24) in Hariana and Karan Swiss cattle, respectively. Ambhore et al. (25) observed genetic correlations of Total LTMY with other lifetime traits, viz., HL and PL as high and positive, whereas between Total LTMY and BE was positive and low in cows. Chaudhari (8) also indicated a positive genetic correlation for Total LTMY with HL and PL; PL with HL and UD. On the contrary, UD with HL and Total LTMY was high and positive in Nili-Ravi buffaloes.

AFC is one of the essential traits for bringing improvement in milk production. It considerably affects the productive life and lifetime milk production of an animal. Reduction in AFC is desirable for reducing rearing cost of heifer and for economizing milk production (5). In agreement with our findings, Chaudhari (8) reported that AFC had positive genetic correlations with HL but negative correlation with UD; FLTMY had moderate to high and positive genetic correlation with Total LTMY and PL. Low and negative genetic correlation of FDP with Total LTMY and HL but positive with UD was also seen. The genetic correlation of FSP was positive with UD and HL and that of FCI was low and positive with Total LTMY, PL, HL, and UD in Nili-Ravi buffaloes. The results are also in line with findings of Ali et al. (4) where significant (*p* ≤ 0.05) and negative genetic correlation of AFC with Total LTMY, PL, HL, and PD was noticed. Negative and significant (*p* ≤ 0.05) genetic correlation between AFC and LTMY has also been reported by Lin and Allaire (26), Bhatia (27), and Hussain (28). Contradictory to our results, Chaudhari (8) reported AFC had low and positive genetic correlations with Total LTMY and PL; low and negative genetic correlation of FDP with PL; genetic correlation of FSP was low and negative with Total LTMY and PL. Genetic correlations of FLTMY with Total LTMY and FDP with BE were low in Murrah buffaloes while high in Nili-Ravi, which may be attributed to variations in genetic makeup of the two breeds.

Our results of positive phenotypic correlation of FLTMY with FLSMY, FPY, and FLL agreed with Chaudhari (8). The phenotypic correlation of FLSMY was high and significant (*p* ≤ 0.05) with FPY and FLL. Similar findings were reported between FLTMY and FLL by Singh and Raut (29) and for FLTMY and FLL in dairy cattle by Samoilo (30). The negative association between FLTMY and FDP could be due to an increase in lactation length or vice versa. Likewise, Patil et al. (22) reported that phenotypic correlation of AFC was significant (*p* ≤ 0.05) and positive with FPY; FSP was non-significantly (*p* > 0.05) negative with FPY; FCI was highly significant (*p* ≤ 0.01) and positive with FLTMY but non-significantly (*p* > 0.05) negative with FPY. In contrast, Dev et al. (31) observed positive phenotypic correlations of FPY with other early performance traits, viz., FSP and FCI. Negative phenotypic correlation of FLSMY with FDP was reported by Chaudhari (8).

High correlation among different lifetime traits illustrates that selection based on any one of these traits would lead to a positive correlated response in other traits. In line with our results, Chaudhari (8) noticed that the phenotypic correlation of Total LTMY was highly significant (*p* ≤ 0.01) with HL, PL, and UD; and of PL was highly significant (*p* ≤ 0.01) with HL and UD. The association observed between UD and HL was also highly significant (*p* ≤ 0.01) in Nili-Ravi buffaloes. Our results also agreed with that of Ambhore et al. (32), where phenotypic correlations of Total LTMY with HL and PL were positive and significant (*p* ≤ 0.05) while with BE was positive and non-significant (*p* > 0.05).

Chaudhari (8) in Nili-Ravi buffaloes reported that phenotypic correlations of AFC were positive with HL; low and negative with UD; FLTMY was positive with PL, HL, and Total LTMY; FDP was low and negative with HL and Total LTMY. The correlations of FSP with HL, PL, UD, and Total LTMY were low and positive; and FCI was low and positive with HL, PL, UD, and Total LTMY. Supportive findings are also reported by Larson et al. (33), Dutt and Taneja (34), and Ali et al. (4) who observed a negative phenotypic correlation between AFC and Total LTMY. On the contrary, Chaudhari (8) found positive phenotypic correlations of AFC with PL, UD, and Total LTMY; negative correlations of FDP with PL and UD. Keeping in view the results, the selection on lifetime performance is not practically feasible due to long generation interval; it is desirable to select the animals on the basis of performance of earlier lactations rather than traits expressed later in life (8).

In conclusion, heritability estimates were high for FPY and BE; moderate for FLTMY and FLL; medium for FLSMY, FDP, FSP, FCI, AFC, and UD. Higher heritability of the traits indicates sufficient additive genetic variance; hence, these traits should be given more attention for selection and breeding strategies to increase genetic gain in the herd. The genetic correlation in Nili-Ravi for AFC was positive with HL and negative with lifetime traits (PL, PD, UD, Total LTMY, Standard LTMY, and BE). Positive genetic correlation for AFC with HL indicates that a delay in first calving offered more time to these animals to stay longer in the herd in unproductive stage until first calving, which might increase herd life of the animals. Negative genetic association of AFC with lifetime traits suggests an animal owner or breeder to make efforts for the reduction of AFC up to a certain extent by means of improved management systems (breeding, feeding, reproduction and housing management, etc.) to enhance production, reproduction, and lifetime performance of the livestock population.

## Data Availability Statement

Data shall be made available as requested by the corresponding authors, without undue reservation.

## Ethics Statement

The study was reviewed and approved by the Institute Animal Ethics Committee, ICAR-Central Institute for Research on Buffaloes, Hisar, India.

## Author Contributions

PT contributed to the collection of data, data curation, and drafting of the manuscript. AB contributed to the design of the experiment. AC contributed to the collection of data and curation. YB contributed to the statistical analysis. AJ contributed to the interpretation of results and revision of the manuscript. All authors contributed to the article and approved the submitted version.

## Conflict of Interest

The authors declare that the research was conducted in the absence of any commercial or financial relationships that could be construed as a potential conflict of interest.
